# *Streptococcus suis* TrpX is part of a tryptophan uptake system, and its expression is regulated by a T-box regulatory element

**DOI:** 10.1038/s41598-022-18227-3

**Published:** 2022-08-17

**Authors:** Muriel Dresen, Desirée Schaaf, Jesús Arenas, Astrid de Greeff, Peter Valentin-Weigand, Andreas Nerlich

**Affiliations:** 1 Institute for Microbiology, University of Veterinary Medicine Hannover, Hannover, Germany; 2 Unit of Microbiology and Immunology, Faculty of Veterinary, University of Zaragoza, Zaragoza, Spain; 3 Wageningen Bioveterinary Research, Part of Wageningen University and Research, Lelystad, The Netherlands; 4 Present Address: Veterinary Centre for Resistance Research (TZR), Freie Universität Berlin, Berlin, Germany

**Keywords:** Microbiology, Bacteriology, Pathogens

## Abstract

*Streptococcus suis*, a common member of the porcine respiratory microbiota, can cause life-threatening diseases in pigs as well as humans. A previous study identified the gene *trpX* as conditionally essential for in vivo survival by intrathecal infection of pigs with a transposon library of *S. suis* strain 10. Here, we characterized *trpX,* encoding a putative tryptophan/tyrosine transport system substrate-binding protein, in more detail. We compared growth capacities of the isogenic *trpX*-deficient mutant derivative strain 10∆*trpX* with its parent. Growth experiments in chemically defined media (CDM) revealed that growth of 10∆*trpX* depended on tryptophan concentration, suggesting TrpX involvement in tryptophan uptake. We demonstrated that *trpX* is part of an operon structure and co-transcribed with two additional genes encoding a putative permease and ATPase, respectively. Bioinformatics analysis identified a putative tryptophan T-box riboswitch in the 5′ untranslated region of this operon. Finally, qRT-PCR and a reporter activation assay revealed *trpX* mRNA induction under tryptophan-limited conditions. In conclusion, our study showed that TrpX is part of a putative tryptophan ABC transporter system regulated by a T-box riboswitch probably functioning as a substrate-binding protein. Due to the tryptophan auxotrophy of *S. suis*, TrpX plays a crucial role for metabolic adaptation and growth during infection.

## Introduction

*Streptococcus suis* is a commensal of the porcine upper respiratory tract but it can also cause life-threatening diseases with symptoms ranging from meningitis, pneumonia, arthritis, and endocarditis to acute sepsis. Therefore, this pathogen represents an economic threat to pig farming worldwide (reviewed in^1,2^). Furthermore, *S. suis* plays an important role as a zoonotic agent causing meningitis and sepsis in human patients^3,4^. The bacterium is genetically highly diverse, as evidenced by the existence of more than 700 sequence types identified by multilocus sequence typing (MLST). Additionally, based on its capsular polysaccharides, *S. suis* can be classified into up to 33 serotypes^5^. Although most of the infections in swine and human are caused by serotype 2 strains^1^, porcine infections by strains of other serotypes, e.g., serotype 9, are becoming increasingly important, particularly in Western Europe^1,6^. In the last decade, an increase of antimicrobial resistance has been described for *S. suis*^7,8^, underlining the urgent need for new therapeutics. Notably, bacterial ATP-binding cassette (ABC) transporters might be interesting targets for drug and vaccine development^9^.

ABC transporters represent a transporter family that is conserved in both eukaryotes and prokaryotes^10^, and play an important role in the survival and virulence of bacteria^11^. This family comprises exporters as well as importers, of which the latter ones are only found in prokaryotes^12^. These importers use a broad range of substrates such as carbohydrates, metals, peptides, amino acids, and others^10^. Typically, ABC transporters consist of two transmembrane domains forming the translocation channel, and two nucleotide-binding domains hydrolyzing ATP for the energy-dependent transport. Some ABC transporters additionally contain a substrate-binding protein that ensures substrate-specificity^11,13^. ABC importers, especially amino acid transporters, are crucial to bacterial survival (biological fitness) in the host. Thus, they are often referred to as virulence-associated factors^11,14,15^. To ensure sufficient nutrient uptake, expression and transport activity of these transporters need to be strictly regulated^11^. Some ABC transporters are regulated by so-called T-box riboswitches^16^. T-box riboswitches are cis-regulatory elements having tRNA as their effector molecule^17^. The riboswitch monitors the aminoacylation status of the tRNA to induce expression of the regulated downstream genes^18^. Thereby, T-box riboswitches can control transcription or translation of the respective genes^19,20^. When the uncharged cognate tRNA binds to a T-box riboswitch, transcription can proceed through stabilization of the antiterminator structure. On the contrary, binding of charged cognate tRNA leads to the formation of the terminator secondary structure and prevents transcription of the regulated gene^17^. The Watson–Crick base pairing between the T-box riboswitch and the tRNA in two critical regions dictates the T-box binding specificity^21^. The specifier sequence, a three-nucleotide sequence in the stem I domain of the T-box riboswitch, interacts with the tRNA anticodon^18^. In addition, the acceptor arm of the uncharged tRNA base pairs with the T-box sequence of the antiterminator bulge^22^. As the antiterminator and terminator conformation are mutually exclusive, the stabilization of the antiterminator prevents formation of the terminator helix^23^. The specifier sequence is specific to one amino acid only^18^. Moreover, the T-box system monitors the ratio of uncharged and charged tRNA rather than its absolute levels^23^.

Using intrathecal infection of pigs with a transposon library of *S. suis*, the gene locus SSU1307 was recently identified as conditionally essential for survival in cerebrospinal fluid and blood meaning a lower growth as compared to THB medium^24^. The protein encoded by this gene shows homologies with a putative tryptophan/tyrosine transport system substrate-binding protein of *Streptococcus pneumoniae*. Therefore, the aim of this study was to analyze the function of SSU1307 in the tryptophan transport process in *S. suis*. Since our previous studies showed that *S. suis* strain 10 is auxotrophic for tryptophan^25^, it is a good model system for analyzing its putative transport via this protein. The putative tryptophan transporter will subsequently be referred to as TrpXYZ analogous to tryptophan ABC transporters in other Streptococci^26^. Accordingly, TrpX represents the putative substrate-binding protein, TrpY the putative permease protein and TrpZ the putative ATPase protein.

## Material and methods

### In silico analysis

SSU1307 and homologous sequences were extracted from the GenomeNet KEGG database and aligned with T-Coffee^27^. The alignment was subsequently visualized using TEXshade^28^. 3D-homology modeling of the protein was done using the SWISS-MODEL server^29^. The server identified protein A0A0H2UPV6 of *S. pneumoniae* (pdb code: 3lft) as best matching template which was used for subsequent homology modelling. Alignment, calculation of the RMSD value, and visualization of the structures were performed with PyMOL 2.5.0a0 (Schrödinger LLC).

Identification of putative tryptophan T-box riboswitches was performed with T-box scan^21^ (https://tbdb.io/tools/tbox-scan.html) with minor modifications (see Github repository mentioned in "Data availability" for details). Verification of transcription terminators in the T-box region and prediction of these sequences at the end of genes was done with ARNold (http://rssf.i2bc.paris-saclay.fr/toolbox/arnold/index.php). The resulting predicted fold of the riboswitch was visualized with R2R 1.0.6^30^. In silico promoter prediction of the *trpX* operon was done with Softberry BPROM software^31^.

### Bacterial strains

The virulent *S. suis* serotype 2 strain 10 was kindly provided by H. Smith (Lelystad, the Netherlands)^32^. Its isogenic *trpX*-deficient mutant, hereafter called 10∆*trpX*, was constructed by inserting a spectinomycin cassette in the corresponding gene (locus tag in strain 10: GPW51_RS06655). DNA fragments were amplified by PCR using primers described in Suppl. Table S1 online. The generation of mutants in strain 10 has already been described^24^. In brief, the spectinomycin-resistance cassette and the flanking regions of *trpX* were amplified from genomic DNA of strain 10. In a new PCR reaction, the cassette was fused to DNA segments of the flanking genes. Finally, these PCR products were transformed in strain 10 using a competence peptide protocol to stimulate transformation^33^. Resulting transformants were selected on Todd-Hewitt Broth (THB; Bacto, Becton Dickinson, Franklin Lakes, NJ, USA) agar plates supplemented with 100 µg/mL spectinomycin (Sigma-Aldrich, St. Louis, MO, USA) and tested by PCR. Correct mutants were confirmed by partial sequencing. The complement strain, hereafter called 10 cTrpX, was constructed by the insertion of a pGA14 plasmid^34^ containing *trpX* and a chloramphenicol cassette in 10∆*trpX*. *TrpX* and its promoter region were amplified by PCR with Q5 polymerase (NEB, New England Biolabs, Ipswich, MA, USA) from genomic DNA of strain 10. Additionally, the plasmid pGA14 already containing a chloramphenicol-resistance cassette was amplified by PCR. Amplified sequences were cloned into *Escherichia coli* DH5α (Thermo Fisher Scientific, Waltham, MA, USA) via in vivo assembly cloning^35^. *E. coli* transformants carrying the plasmid were identified by PCR, confirmed by partial sequencing, and used to transform 10∆*trpX* as previously described^33^. Transformants were selected on THB agar supplemented with 4 µg/mL chloramphenicol (Carl Roth, Karlsruhe, Germany).

The reporter plasmid was constructed by the insertion of the *trpX* promoter region and 5′-UTR in a pGA14 plasmid carrying a green fluorescent protein (GFP) sequence as well as a spectinomycin resistance-cassette. The promoter region was amplified from genomic DNA of strain 10. The plasmid backbone was amplified separately. Amplified sequences were cloned into *E. coli* DH5α via in vivo assembly cloning as described above. Plasmids were confirmed by sequencing and transformed in strain 10 as previously described^33^. The resulting strain was called 10::*trpXYZ*-prom-*gfp*. Different mutations of conserved T-box regions were introduced into the reporter plasmid. We deleted the stem I domain and mutated the specifier sequence as well as the T-box sequence by site-directed mutagenesis. Besides, we deleted the complete T-box riboswitch sequence. Mutations and deletions were generated by using for this purpose designed primer pairs according to Garcia-Nafria et al*.*^35^. PCR, ligation, and transformation were performed as described for the original reporter plasmid.

### Growth experiments in chemically defined medium

Bacterial strains were cultured overnight at 37 °C on Columbia agar supplemented with 7% (v/v) sheep blood (strain 10 and 10Δ*trpX*) (Thermo Fisher Scientific) or on THB agar (Becton Dickinson) supplemented with 4 µg/mL chloramphenicol (strain 10 cTrpX) under aerobic conditions. For each experiment, a starting culture was inoculated with two to three single colonies in THB medium supplemented with the corresponding antibiotic and incubated at 37 °C overnight. The next day, bacterial cultures were adjusted to an optical density at 600 nm (OD_600_) of 0.02 in THB medium with (10 cTrpX) or without antibiotics and incubated to an OD_600_ of 0.5. Then, 5 mL of each strain were pelleted (10 min, 4696×*g*, 4 °C) and washed with 4 mL chemically defined basal medium (CDM). CDM was prepared as previously described with modifications in tryptophan concentrations^25^ (Suppl. Table S2 online). Next, the bacteria were resuspended in 5 mL CDM basal medium and subsequently adjusted to an OD_600_ of 0.02 in full CDM containing different tryptophan concentrations. In a second experimental setup, different concentrations of either a tryptophan- or a tyrosine-tripeptide (Bachem, Bubendorf, Switzerland) were added to CDM containing 5 mg/mL tryptophan. The concentration of 5 mg/L tryptophan was chosen as a reference for further experiments as it was shown to be sufficient for normal growth of the wild-type strain, but not for 10Δ*trpX*. OD_600_ measurements were performed in triplicate in a 96-well-plate covered with a Breathe-Easy sealing membrane. Absorbance at 600 nm was measured automatically every 30 min in a SpectraMax i3x plate reader (Molecular Devices, San Jose, CA, USA) for 24 h at 37 °C. The plate was shaken before each measurement.

### Purification and analysis of RNA transcripts

*S. suis* strain 10 was prepared as described in “Growth experiments in chemically defined medium”. Subsequently, bacterial cultures were adjusted to an OD_600_ of 0.02 in CDM containing four different tryptophan concentrations. Suitable concentrations were determined by a growth experiment of strain 10 in CDM under tryptophan-limited conditions. Bacteria were grown to an OD_600_ of 0.5 (reference: medium with 5 mg/L tryptophan), harvested by centrifugation, resuspended in 1 mL ice-cold TRI Reagent (Zymo Research, Irvine, CA, USA), disrupted by a FastPrep-24 5G Instrument (3 × 45 s, speed 6.5) (MP Biomedicals, St. Ana, CA, USA) and cooled on ice. RNA was purified using the Direct-zol RNA Miniprep Kit (Zymo Research) in accordance with the manufacturer’s recommendations including DNase treatment. On-column DNA digestion was performed with the Qiagen DNase (Qiagen, Venlo, the Netherlands). An additional DNase treatment was performed after RNA isolation with the RNeasy Mini Kit (Qiagen). qRT-PCR was performed as previously described^36^. Briefly, 2 µg of total RNA were reverse transcribed with 500 ng of Random Primers (Promega, Madison, WI, USA) and 200 units of M-MLV RT (Promega). The cDNA samples were diluted 1:20 in nuclease-free water and stored at − 20 °C. The qRT-PCR reactions were performed with SYBRGreen Mix (Qiagen) and the Stratagene Mx3005P system (Agilent, St. Clara, CA, USA) and primer pairs indicated in Suppl. Table S1 online. ‘No template’ and ‘no-RT’ controls were included in all runs. Expression levels of tested genes were normalized using the *dnaH* gene of *S. suis* as an internal standard. The best-suiting housekeeping gene (*dnaH*) was determined with BestKeeper software^37^ by testing three different genes (*gyrB*, *dnaH, mapZ*). Amplification﻿ efficiency was determined for each primer pair using serial dilutions of pooled cDNA. Gene expression was calculated by the ΔC_t_ method^38^.

### Operon analysis, 5′ rapid amplification of cDNA ends and rapid amplification of cDNA ends from circularized RNA

Strain 10 was prepared as described in “Growth experiments in chemically defined medium” and harvested at an OD_600_ of 0.5. RNA isolation and cDNA synthesis was performed as described in “Purification and analysis of RNA transcripts”. By combining different primer pairs (Suppl. Table S1 online) the promoter region was characterized by RT-PCR. PCR reactions were conducted with HotStarTaq Plus DNA polymerase (Qiagen). Five prime rapid amplification of cDNA ends (5′ RACE) was performed using the Template Switching RT Enzyme Mix (NEB) in accordance with the manufacturer’s recommendations. Rapid amplification of cDNA ends from circularized RNA (cRACE) was performed according to Brenneis et al.^39^. For this, RNA of *S. suis* strain 10 isolated from CDM containing 5 mg/L tryptophan (see “Purification and analysis of RNA transcripts”) was used. First, the pyrophosphate from the 5′ triphosphorylated RNA was removed using RppH (NEB) in accordance with the manufacturer’s recommendations. Second, RNA was circularized with T4 RNA ligase (NEB) and RNase inhibitor (Thermo Fisher Scientific) was added to the reaction. RNA cleanup was performed using the Monarch RNA Cleanup Kit (NEB). Subsequently cDNA synthesis was performed as described in “Purification and analysis of RNA transcripts” but with a gene-specific reverse primer instead of random primers. The 3′ and 5′ end were then amplified by two nested PCRs. The resulting amplicon was purified with the Monarch DNA Cleanup Kit (NEB) and analyzed by sequencing.

### Fluorescence microscopy and flow cytometry analysis

Bacteria were prepared as described in “Growth experiments in chemically defined medium”. Subsequently, the bacterial cultures were adjusted to an OD_600_ of 0.02 in full CDM containing four different tryptophan concentrations and 100 µg/mL spectinomycin. Bacteria were grown to an OD_600_ of 0.5 (reference: strain 10 in medium with 5 mg/L tryptophan). For fluorescence microscopy, 2 µL of the bacterial cultures were spotted on 1% low-melting agarose (GERBU, Heidelberg, Germany) pads. Samples were analyzed using a Nikon Eclipse Ti-S microscope (Nikon, Tokyo, Japan), equipped with the objective Plan Fluor 100 ×/1.3 oil. Images were identically adjusted for brightness and contrast in ImageJ/Fiji^40^.

Additionally, 250 µL of bacterial suspension were fixed with formaldehyde (Polysciences, Warrington, PA, USA; final concentration of 3%) and stored at 4 °C. The next day, samples were pelleted (1690×*g*, 10 min), and resuspended in 500 µL sterile-filtered (0.22 µm) PBS for flow cytometry analysis. GFP signal was measured using Guava EasyCyte8 (Merck Millipore, Darmstadt, Germany). The cell population was identified using forward and side scatter. Non-fluorescent and green-fluorescent cells were detected. In each experiment, more than 30,000 cells were analyzed. The results were analyzed with FlowJo software version 10.8.1 (Tree Star Inc., Ashland, OR, USA). For this, bacteria were identified using forward and side scatter. The Downsample plugin was used to create a representative subpopulation of each sample which was then used for further analysis (http://docs.flowjo.com/d2/plugins/downsample/). Each experiment was repeated at least three times. Data are displayed as histograms and median fluorescence intensity (MFI). The gating strategy for FlowJo analysis is shown in Suppl. Fig. S1 online.

### Statistical analysis

Values are expressed as means ± SD. Sample size is given in figure legends. Analysis using estimation statistics was done with Python 3.9 (Python Software Foundation, https://www.python.org/) and the DABEST package v0.3.1^41^. Generated Cumming plots display the magnitude and robustness of the effect size and its bootstrapped 95% confidence interval (95% CI).

## Results

### In silico analysis of *S. suis* TrpX

**Figure 1:**
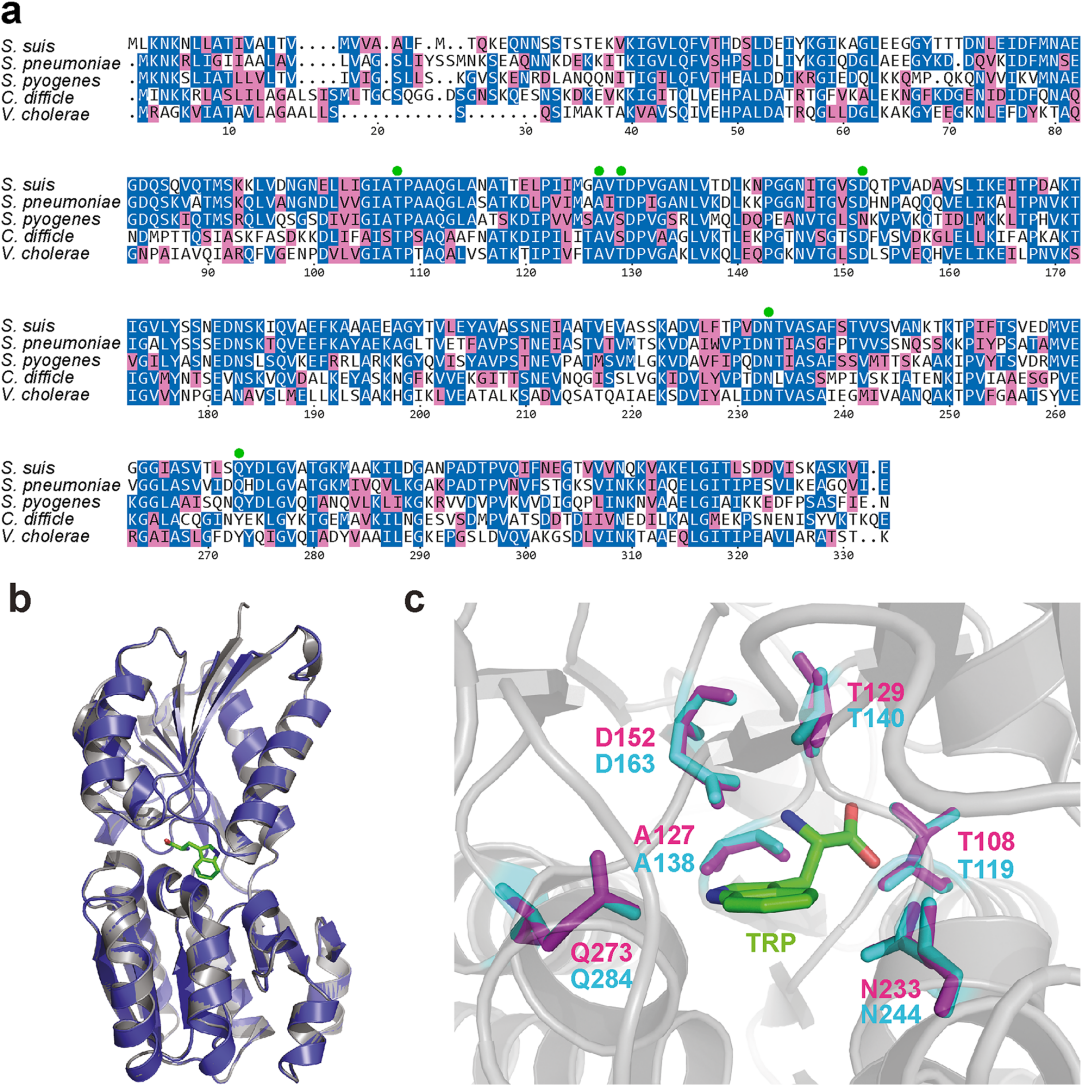
Comparative in silico analysis of TrpX protein with homologs of other bacterial species. (**a**) Alignment of the amino acid sequences of the protein encoded by SSU1307 (locus tag in P1/7 genome) in *S. suis* strain 10 (locus tag: GPW51_RS06655) and the homologous proteins of *S. pneumoniae* (UniProtKB A0A0H2UPV6), *S. pyogenes* (UniProtKB Q99ZY6), *C. difficile* (UniProtKB Q18A65) and *V. cholerae* (UniProtKB Q9KT04). Identical amino acids are shown in blue, amino acids with a similar function are marked in light pink. Green dots indicate amino acids important for substrate-binding. (**b**) Comparison of the in silico generated structure of TrpX (amino acids 40–332) with the crystal structure of A0A0H2UPV6 (pdb: 3lft). The computed structure of TrpX is shown as blue cartoon, whereas the reference structure is shown as grey cartoon. The ligand of A0A0H2UPV6, tryptophan, is shown in green. (**c**) Comparison of amino acids involved in substrate binding in A0A0H2UPV6 in cyan and the model in magenta. Tryptophan is shown in green and 3lft as cartoon in grey.

The gene encoding the putative tryptophan transporter substrate-binding protein TrpX of strain 10 had been previously identified as conditionally essential for bacterial survival in cerebrospinal fluid and blood of pigs^24^. Alignment of the predicted TrpX protein with homologous proteins in other streptococci revealed an overall homology of 59% with *S. pneumoniae* (UniProtKB A0A0H2UPV6) and of 45% with *Streptococcus pyogenes* (UniProtKB Q99ZY6) (Fig. 1a). Besides, the protein is present in other bacterial species (Fig. 1). Bradshaw et al*.* identified several amino acids of this protein that are important for ligand binding^42^ and are highly conserved in the different species (green dots, Fig. 1a). Two of these amino acids are of special importance for substrate specificity and vary between species, possibly due to different substrate preferences^42^ (Fig. 1).

Using the available structural data of the *S. pneumoniae* protein A0A0H2UPV6 (pdb code: 3lft) we established an in silico model of the structure of TrpX by employing the SWISS-MODEL server^29^. Superposition of the model and the structure of A0A0H2UPV6 revealed that TrpX has the same fold and domain structure as the pneumococcal substrate binding protein with an RMSD value of 0.443 Å (Fig. 1b). We also compared amino acids involved in ligand binding within pneumococcal protein A0A0H2UPV6 with those found in TrpX and observed positional conservation of these amino acids in both structures (Fig. 1c). In particular, A127, T108, T129, D152, and N233 of TrpX have identical counterparts in the analyzed substrate-binding proteins, except for *S. pyogenes* which contains the asparagine instead of aspartate (position D152 in TrpX). Q273 is only conserved in the three streptococcal proteins but not in the *Clostridioides difficile* and *Vibrio cholerae* protein and might therefore be important for substrate specificity. Since amino acids involved in ligand binding are conserved in the different substrate binding proteins especially in those of the streptococcal species, and due to the fact that the pneumococcal homolog binds tryptophan, we subsequently performed growth experiments with the 10∆*trpX* mutant in comparison with the parent strain 10 in CDM containing different tryptophan concentrations.

### Growth in CDM reveals a tryptophan-dependent growth defect of 10Δ*trpX*

**Figure 2:**
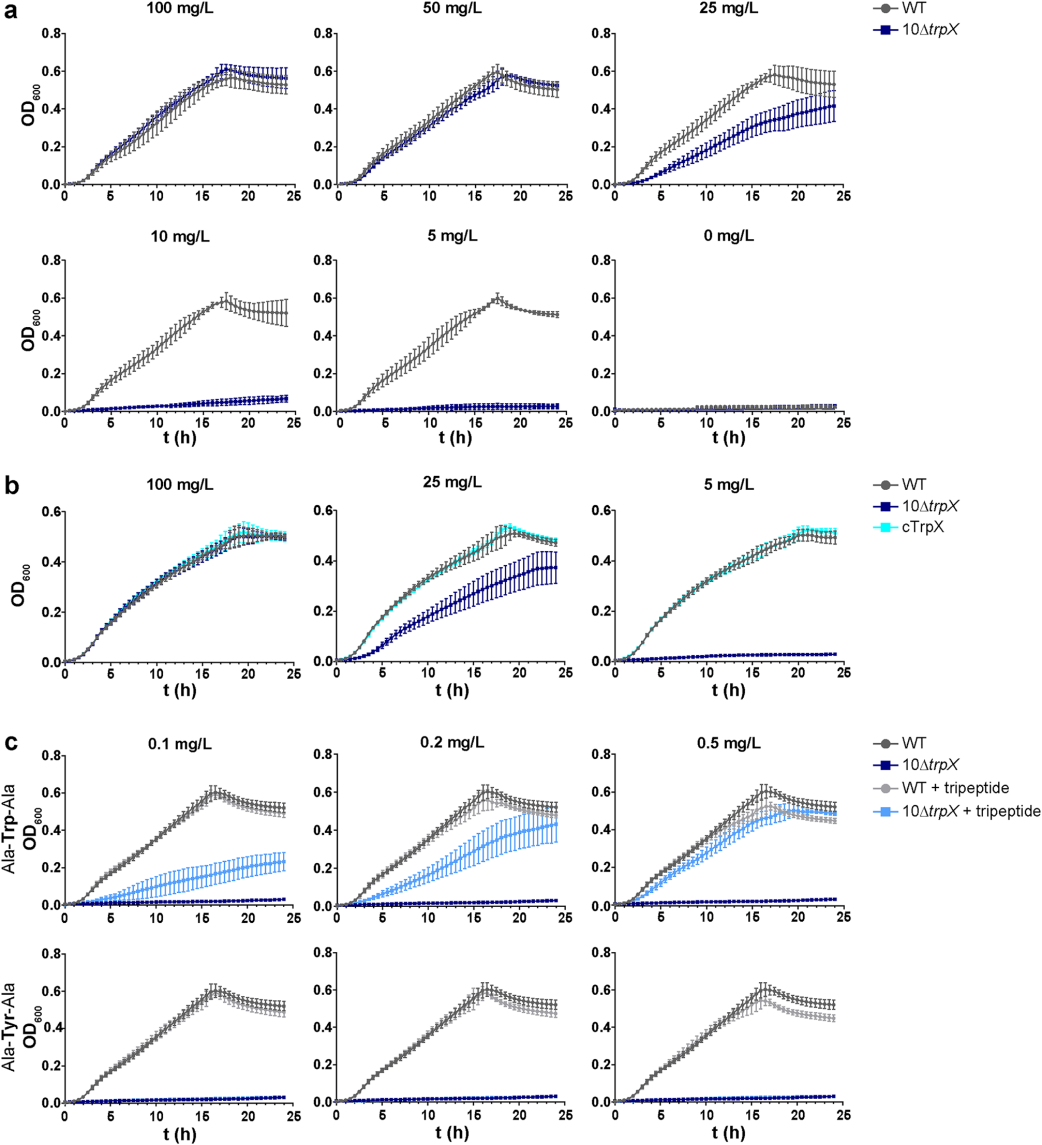
Growth in CDM. *S. suis* strain 10 (grey), 10Δ*trpX* (blue) and 10 cTrpX (cyan) were grown in CDM for 24 h and the OD_600_ was determined every 30 min. Data are presented as mean ± SD of four independent experiments. (**a**) *S. suis* strain 10 and 10Δ*trpX* grown in CDM supplemented with different tryptophan concentrations. (**b**) Growth of strain 10, 10Δ*trpX*, and 10 cTrpX in CDM with different tryptophan concentrations. (**c**) Strain 10 and 10Δ*trpX* grown in CDM with 5 mg/L tryptophan and supplemented with different concentrations of either a tryptophan- or a tyrosine-tripeptide. Growth of the strains in CDM complemented with tripeptides is shown in the corresponding light color.

TrpX is predicted to bind tryptophan. To test this hypothesis, we compared the growth of strain 10∆t*rpX* with that of the wild-type strain in CDM containing different tryptophan concentrations. At a concentration of 100 mg/L tryptophan, which is the standard concentration in CDM for *S. suis*^25^, 10Δ*trpX* showed a comparable growth as the parent (Fig. 2a). At moderate tryptophan concentrations, e.g., at 25 mg/L, the growth of the mutant was reduced. At low concentrations (10 mg/L and below), the growth of 10Δ*trpX* was nearly completely abolished. In CDM without tryptophan, neither the wild type nor the mutant was able to grow (Fig. 2a), which confirmed the previously described tryptophan auxotrophy of *S. suis* strain 10^25^.

Next, we complemented the observed phenotype by reintroducing the *trpX* gene in 10Δ*trpX* via a plasmid, resulting in strain 10 cTrpX*.* Growth experiments of 10 cTrpX in CDM with three different tryptophan concentrations revealed that the complementation restored the growth defect of the mutant (Fig. 2b). This showed that the observed phenotype was caused by the deletion of *trpX*.

The concentration of 5 mg/L tryptophan was chosen as a reference for further experiments as it was shown to be sufficient for normal growth of the wild-type strain, but not for 10Δ*trpX*. We assumed that the impaired growth of the mutant was due to an insufficient uptake of tryptophan. Thus, we next supplemented the media with different tripeptides. Peptides composed of amino acids required by respective auxotrophic bacteria can satisfy their nutritional needs^43^. We used a tryptophan-tripeptide and included a tyrosine-tripeptide as a control. Tripeptides are taken up via special peptide transporters. Inside the cell, peptides are cleaved, thereby making single amino acids available to the bacterium^43^. We hypothesized that adding a tryptophan-tripeptide would allow the growth of the mutant in CDM with low tryptophan concentrations since these peptides will be taken up by other peptide transporters, e.g. the oligopeptide transporter OppA of *S. suis*^44^. The addition of different tryptophan-tripeptide concentrations revealed a dose-dependent increase of 10Δ*trpX* growth, whereas the addition of the tyrosine-tripeptide showed no effects (Fig. 2c). The growth characteristics of the wild type were not affected by the supplementation with tripeptides. In summary, these results suggest that tryptophan is the limiting factor for the impaired growth of 10Δ*trpX* in CDM.

### *TrpX* is part of an operon

**Figure 3:**
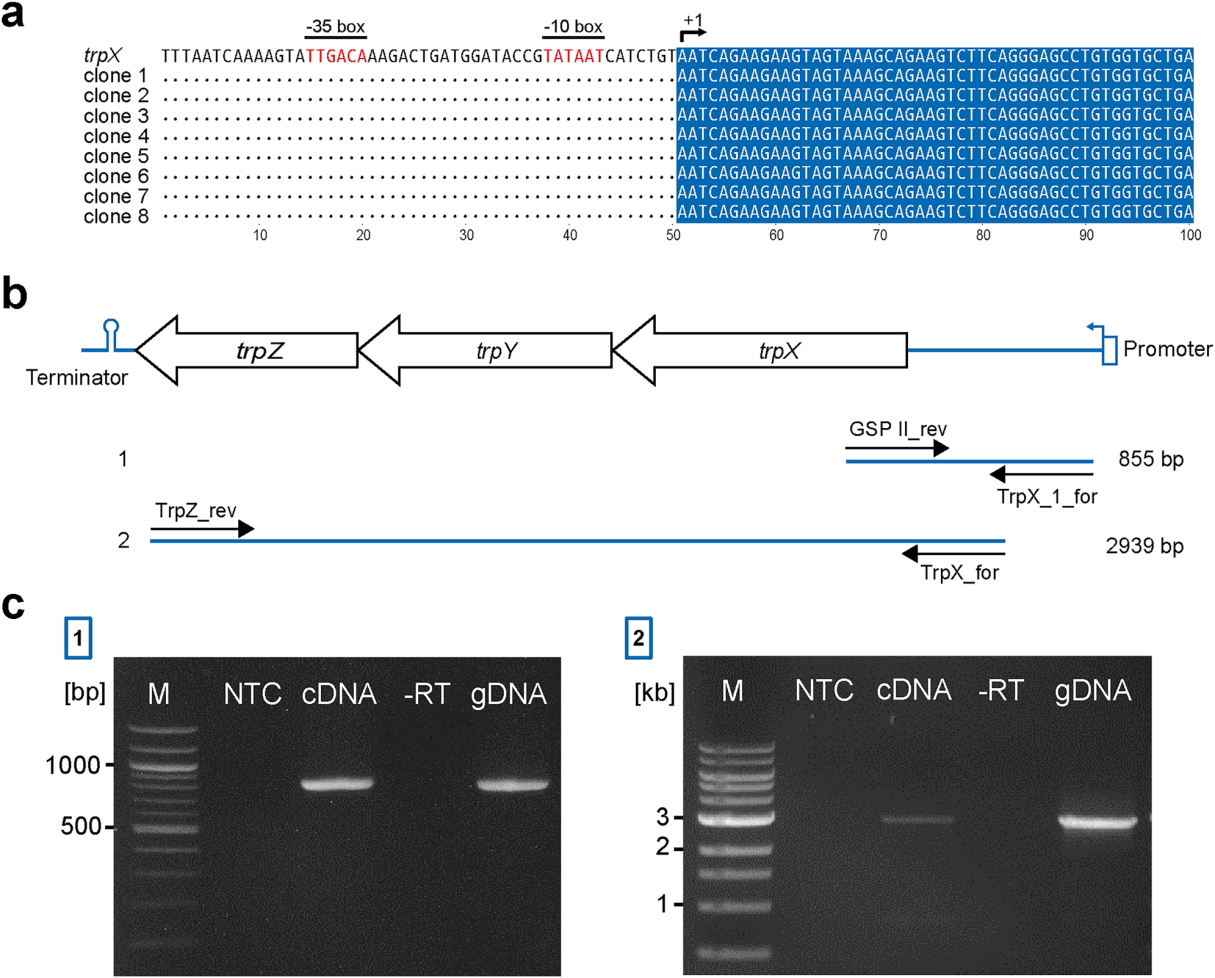
*trpX* is part of an operon. (**a**) Identification of the *trpX* transcriptional start site (TSS) by 5′ RACE. Alignment of the genomic region upstream *of trpX* with clones obtained by 5′ RACE from one representative out of two experiments. Prediction of regulatory elements is highlighted in red. The position of the identified TSS (+ 1) is indicated by an arrow. Dots indicate nonmatching nucleotides. (**b**) Genomic organization of *trpX* to *trpZ* and illustration of possible amplification products with the indicated primer pairs (solid blue lines), scheme not true to scale. (**c**) Corresponding representative agarose gels. *NTC* non-template control, *cDNA* standard RT-PCR reaction using reverse transcribed RNA, *−RT* negative control in which no reverse transcriptase was added to the RT reaction, *gDNA* positive control in which genomic DNA was used as a template in the RT-PCR. Left [1] and right [2] gel show the products obtained with GSP_II_rev and TrpX_1_for and TrpZ_rev and TrpX_for, respectively as depicted in panel (**b**). Original gels are presented in Suppl. Fig. S2 online.

Metabolism regulation plays a key role in bacterial survival and growth in the host. Therefore, respective metabolic genes are often organized as an operon, and their expression is regulated in response to changes in environmental conditions^45^. An operon comprises several genes that are co-transcribed in a single messenger RNA (mRNA) under the control of a shared promoter^46^. Indeed, the chromosomal proximity of *trpX*, *trpY*, and *trpZ* suggests their organization as an operon, which is common in genes coding for particular ABC transporters^47^. To prove this, we first performed in silico promoter prediction analysis using the upstream region between the translational start site (ATG) of *trpX* and the previous gene and obtained three predicted promoters. The one with the highest score was located − 676 bp upstream of the ATG start codon (Suppl. Table S3 online) suggesting the presence of an approximately 680 bp 5′ untranslated region in front of *trpX*. Next, 5′ RACE was performed to experimentally determine the transcriptional start site (TSS). All clones obtained in this experiment confirmed that the TSS is located − 676 bp upstream of the ATG of *trpX* (Fig. 3a). To prove that the leader region of the *trpX* gene is co-transcribed with the *trpX* coding region and forms an operon with *trpY* and *trpZ*, we performed RT-PCR of the resulting cDNA using a combination of specific primer pairs (Fig. 3b). Firstly, we amplified an 855 bp fragment (from − 641 to + 214 bp relative to the TSS) including the non-coding leader sequence as well as the 5′ region of the *trpX* coding region (Fig. 3c). Secondly, we used another primer pair to amplify a 2939 bp fragment (from − 338 to + 2601 bp relative to the TSS) that includes a part of the 5′ untranslated region as well as the coding regions for *trpX*, *trpY* and *trpZ* (Fig. 3c). Results demonstrated that the 5′ untranslated region of the *trpX* gene is co-transcribed with the coding regions of the genes *trpX* to *trpZ*, thus forming an operon that contains all three genes that code for the components of the ABC transporter system.

### *trpXYZ* mRNA contains a T-box riboswitch

**Figure 4:**
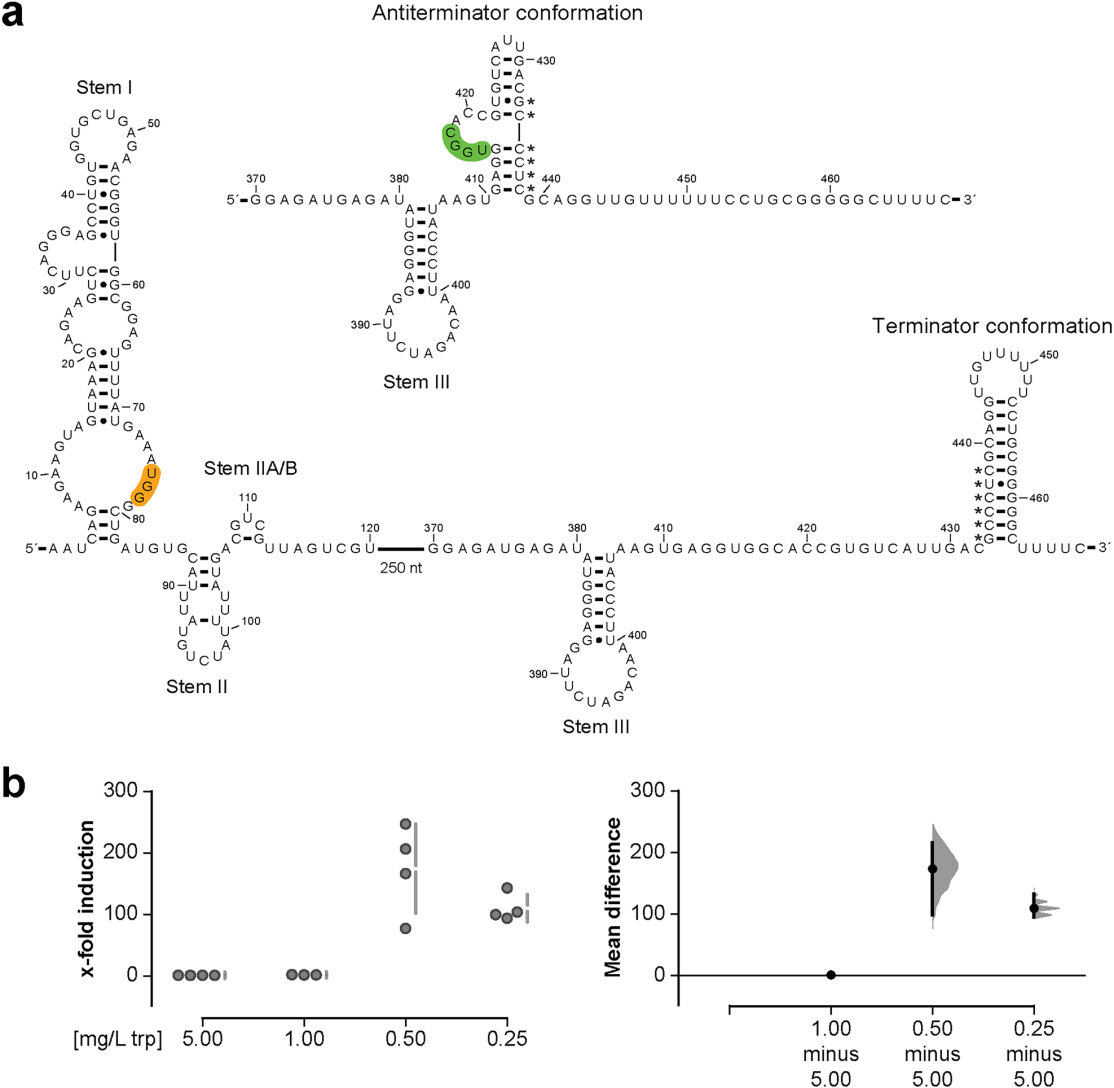
*trpXYZ* mRNA contains a T-box riboswitch. (**a**) Visualization of the predicted *trpX* T-box riboswitch using T-box scan^21^. The specifier sequence that is exclusive for tryptophan is highlighted in orange. The T-box sequence is displayed in green. Stem I, stem II, stem IIA/B and stem III are indicated. Binding of uncharged tRNA to the specifier sequence as well as the T-box sequence results in stabilization of the antiterminator conformation leading to transcription of the downstream gene (upper structure). Binding of charged cognate tRNA to the specifier sequence results in the formation of the terminator secondary structure and terminates transcription (lower structure; 3′ region). Asterisks indicate nucleotides that are involved in both, mutually exclusive structures, the antiterminator and the terminator. (**b**) Quantification of *trpX* mRNA expression under tryptophan-limited conditions from n ≥ 3 experiments. Individual data points and summary measurements (mean ± SD) are plotted in the left panel; effect size (mean differences, black dot) with bootstrapped 95% confidence intervals and resampling distribution are shown in the right panel.

T-box riboswitches are cis-regulatory elements monitoring the aminoacylation status of the tRNA to e.g., induce expression of regulated downstream genes^17,18^. T-box scan in combination with ARNold terminator prediction was used to predict the T-box riboswitch of *trpX* and the homologous genes of *S. pneumoniae* and *S. pyogenes* (Suppl. Table S4 online). This tool applies inference of RNA alignments (INFERNAL) to identify T-box riboswitches and their features based on a sequence input^21^. T-box scan predicted a tryptophan T-box for *S. suis* and *S. pneumoniae*, whereas no hit was found for *S. pyogenes* (Suppl. Table S4 online). The predicted tryptophan T-box riboswitch is 461 bp in length (from position 2 to 463 after the TSS) and contains the typical structures formed within the T-box RNA including stem I, stem II, stem IIA/B, stem III as well as the sequences that can form the terminator/antiterminator structure (Fig. 4 and Suppl. Fig. S3 online). Stem I contains the specifier sequence UGG that can form a codon-anticodon interaction with the tryptophanyl-tRNA. Following stem III, the T-box sequence UGGC is located in the anti-terminator sequence that precedes the transcription terminator sequence in the leader region of the *trpX* gene, suggesting regulation of the operon by transcriptional attenuation. The terminator loop is followed by a stretch of four uridines (U) residues (Fig. 4 and Suppl. Fig. S3 online). In bacteria, intrinsic terminators are usually formed by a G/C-rich hairpin structure followed by a thymine stretch of approximately seven to nine thymines (T) in the DNA or Us in the transcribed RNA^48,49^. Hence, we performed rapid amplification of cDNA ends from circularized RNA (cRACE) to determine the exact 3′ end of the mRNA. cRACE confirmed that transcription terminates one base after the four U-stretch under non-inducing conditions (Suppl. Fig. S4 online). Besides, the transcriptional start site was verified with this method (Suppl. Fig. S4 online).

During tryptophan-limited conditions, the majority of tRNAs are uncharged and can bind to both the specifier and the T-box-sequence, thereby stabilizing the antiterminator loop which then leads to the transcription of the gene. The expression of the transporter enables the import of tryptophan. In an environment with high tryptophan concentrations, the charged tRNA can only bind to the specifier sequence. This leads to the formation of the terminator structure and premature termination of transcription. Thus, we hypothesized that the mRNA of *trpX* is induced under tryptophan-limited conditions. To prove this, we performed growth experiments of strain 10 in CDM with low tryptophan concentrations (Suppl. Fig. S5 online). When the bacteria in the reference concentration (5 mg/L tryptophan) reached an OD_600_ of 0.5, total RNA was isolated, reverse-transcribed and analyzed by qRT-PCR. In total, we tested four different tryptophan concentrations. Bacteria grown in CDM with the two lowest concentrations showed a significant increase of mRNA induction (Fig. 4b), whereas the intermediate concentration of 1 mg/L was comparable to the reference. Although, the wild type already showed reduced growth at a concentration of 1 mg/L tryptophan (Suppl. Fig. S5 online), we could not observe an mRNA induction at this concentration. In summary, low tryptophan concentrations led to an increase in mRNA expression of *trpX*.

### A *trpX* riboswitch-reporter fusion construct is activated at low tryptophan concentrations

**Figure 5:**
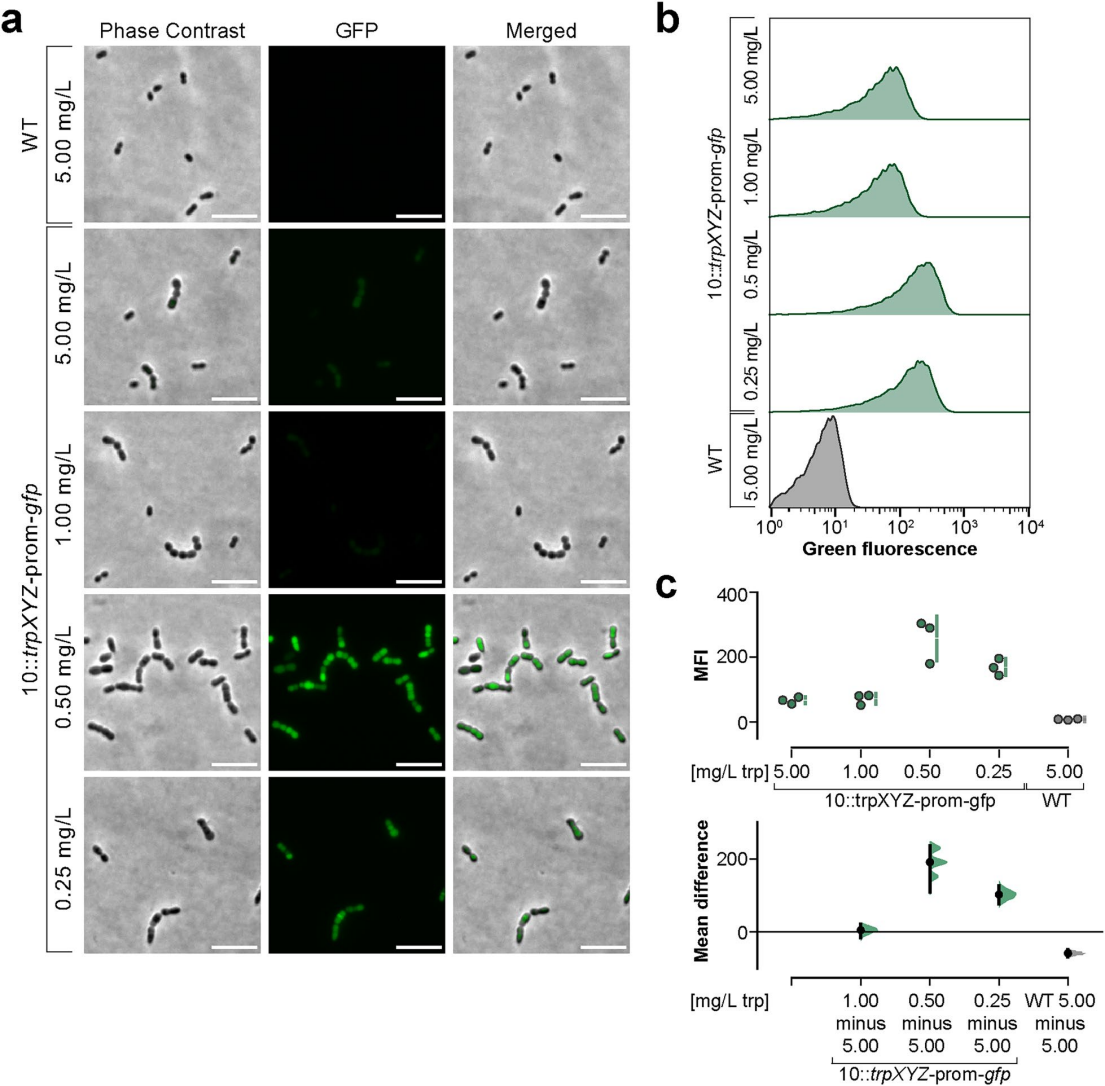
Reporter assay of *trpX* induction. (**a**) Bacterial cultures were grown in CDM containing different tryptophan concentrations (5.00 mg/L, 1.00 mg/L, 0.50 mg/L and 0.25 mg/L) to an OD_600_ of 0.5 and subsequently analyzed by light and fluorescence microscopy. Strain 10 (WT) grown in CDM with 5.00 mg/L tryptophan was used as a control. One representative out of three independent experiments is shown. Scale bars represent 5.00 µm. (**b**) Bacterial cultures were grown in CDM containing different tryptophan concentrations to an OD_600_ of 0.5 and subsequently analyzed via flow cytometry. Fluorescence intensity of the analyzed strains is depicted as histograms. Strain 10 (WT) grown in CDM with 5.00 mg/L tryptophan was used as a negative control (grey). 10::*trpXYZ*-prom-*gfp* (green) was grown in CDM with different tryptophan concentrations as indicated above. One representative histogram out of three independent experiments is shown. In (**c**) the quantification of the median fluorescence intensity (MFI) of the different strains from n = 3 experiments is shown. Individual data points and summary measurements (mean ± SD) are plotted in the upper panel; effect size (mean differences, black dot) with bootstrapped 95% confidence intervals and resampling distribution are shown in the lower panel.

The qRT-PCR revealed an increase in mRNA induction of *trpX* under tryptophan-limited conditions. To further analyze regulation under these circumstances, we constructed a reporter plasmid carrying the promoter and the 5′ non-coding region of *trpX* in front of a *gfp* gene, transformed it into the wild-type strain and determined *gfp* expression in the resulting strain, 10::*trpXYZ*-prom-*gfp*, in CDM using the same tryptophan concentrations as for the qRT-PCR assays. The bacteria were analyzed by light and fluorescence microscopy and by flow cytometry using the wild-type strain as a non-fluorescent control. Investigation by fluorescence microscopy revealed absence of fluorescence in the wild type (Fig. 5a). However, examination of 10::*trpXYZ*-prom-*gfp* grown in CDM revealed increased fluorescence emission inversely related to the concentration of tryptophan in the culture medium (Fig. 5a). Flow cytometry analysis confirmed these results (Fig. 5b). Indeed, the examination of the fluorescence intensity of 10::*trpXYZ*-prom-*gfp* showed low intensity at 5 mg/L and 1 mg/L tryptophan and high intensity at 0.5 mg/L and 0.25 mg/L (Fig. 5b/c). Altogether these investigations confirmed the results of the qRT-PCR and provide further evidence for regulation of gene expression by the putative riboswitch located in the leader region of the *trpX* mRNA.

### Mutation of conserved motifs in the T-box riboswitch affect reporter activation

The reporter assay demonstrated an increase in the transcription of the promoter and the 5′ non-coding region of *trpX* at low tryptophan concentrations. In a next step, we mutated highly conserved domains of the T-box riboswitch, to further prove that the transcription is regulated by the T-box riboswitch. The stem I domain includes the specifier sequence which is important for ligand recognition^50^ as it directly interacts with the tRNA anticodon^18^. Besides, the interaction between Stem I and the elbow region of the tRNA contributes to this binding step^51^.

**Figure 6:**
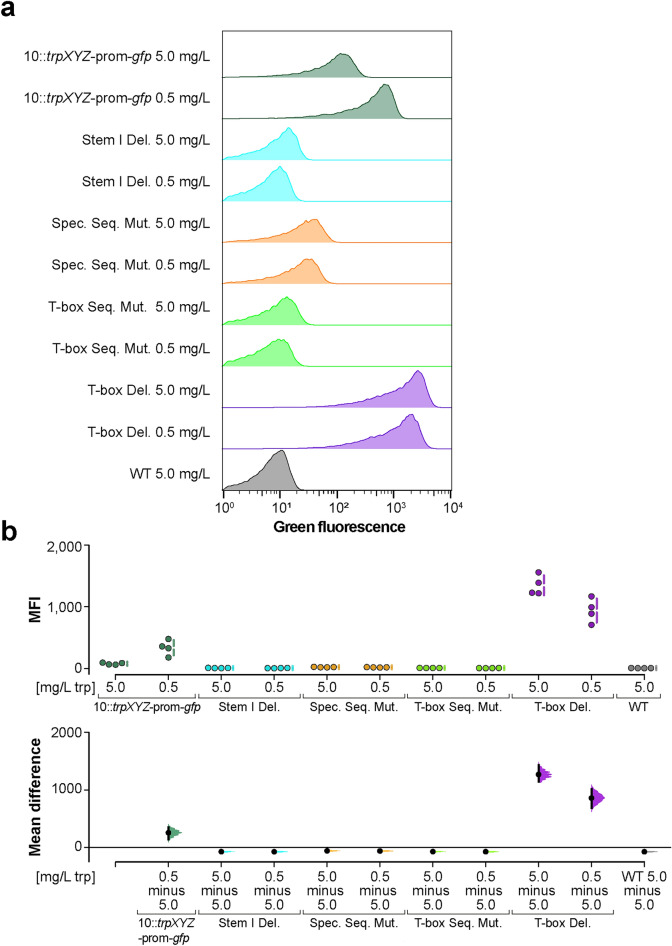
Flow cytometry analysis of *trpX* promoter activity in T-box mutants under tryptophan-limited conditions. Bacterial cultures were grown in CDM containing two different tryptophan concentrations (5.0 mg/L or 0.5 mg/L) to an OD_600_ of 0.5 and subsequently analyzed via flow cytometry. The corresponding histograms are shown in (**a**). In (**b**) the quantification of the median fluorescence intensity (MFI) of the different strains from n = 4 experiments is shown. Individual data points and summary measurements (mean ± SD) are plotted in the upper panel; effect size (mean differences, black dot) with bootstrapped 95% confidence intervals and resampling distribution are shown in the lower panel. Different strains and mutants are indicated by following colors: Strain 10 (WT) is shown in grey, 10::*trpXYZ*-prom-*gfp* in dark green, the deletion of the stem I domain in cyan, the mutated specifier sequence in orange, the mutated T-box sequence in light green, and the deletion of the T-box in purple.

Therefore, we deleted the stem I domain to analyze its influence on reporter activation. Additionally, we mutated the specifier sequence as well as the T-box sequence by site-directed mutagenesis to specifically investigate their role in reporter induction. Finally, we also deleted the complete T-box riboswitch. The four resulting mutants were tested in the reporter assay described above using two different tryptophan concentrations (5.0 mg/L: no induction of the reporter; 0.5 mg/L: induction of the reporter). Subsequently the samples were analyzed by flow cytometry. Results revealed that the deletion of the stem I domain resulted in lack of induction of the reporter under tryptophan-limited conditions. The strains carrying the mutagenized specifier sequence or T-box sequence also showed no increase in fluorescence intensity at 0.5 mg/L (Fig. 6), confirming that they are key motifs of the T-box riboswitch. On the other hand, the deletion of the complete T-box riboswitch led to a significant increase in *trpX* riboswitch-reporter induction independent from tryptophan concentration. Therefore, the absence of the T-box regulator resulted in constitutive activation of the *trpX* riboswitch-reporter.

## Discussion

ABC transporters play a crucial role in pathogen survival in the host and establishment of infection^14,52^. In *Escherichia coli* almost 5% of the genome encodes for ABC transporters^53^. To ensure sufficient nutrient uptake, these transporters require tight regulation^11^. The *S. suis* genome lacks genes encoding for enzymes, which are required for biosynthesis of several amino acids including tryptophan^25^. Due to its tryptophan auxotrophy, TrpXYZ is of special importance for the survival and growth of *S. suis* strain 10 in nutrient-limited environments.

Alignment of the protein sequence of TrpX with homologs of other bacterial species revealed a high homology and prompted us to study if indeed this protein is involved in tryptophan uptake and how its expression is regulated. Besides, the sequence homology of the protein encoded by *trpX* with the pneumococcal orthologue suggested tryptophan as the possible ligand. 3D-homology modeling based on the structure of the *S. pneumoniae* protein confirmed this assumption. The amino acid glutamine at position 273 (Q273), is important for ligand specificity and corresponds with the structure of the pneumococcal protein, which is predicted to bind tryptophan^42^. Thus, we decided to probe that TrpX is indeed part of a tryptophan uptake system by generating a *trpX*-deficient mutant.

Growth experiments in CDM showed a tryptophan-dependent growth defect of 10Δt*rpX*. At low tryptophan concentrations the growth of 10Δ*trpX* was clearly impaired in comparison to the wild-type strain. However, the mutant was able to grow normally in medium with high tryptophan concentrations, suggesting that the transporter is still functional under these conditions or the bacteria may use other transporters with low tryptophan affinity. Similar results were obtained for a homologous mutant in *S. pyogenes*^54^. The substrate-binding protein of an ABC transporter is responsible for conferring high affinity import. In its absence the permease protein may retain substrate-specificity, but with reduced affinity^54^. This phenomenon has also been described in other transport systems, e.g., in *E. coli*^55,56^. Notably, there are also ABC transport systems lacking a substrate-binding protein. In these importers, the transmembrane domain is divided into a substrate-specific and an energy-coupling unit^57^. Thus, a substrate-binding protein is not always required for transport activity. The addition of a tryptophan-tripeptide restored the growth defect of 10Δ*trpX* at low tryptophan concentrations in a dose-dependent manner. Its uptake via peptide transporters bypasses the need for an ABC transporter^54^, i.e. TrpXYZ, as metabolization of the peptide inside the cell seems to provide enough tryptophan for normal growth. Supplementation with an analogous tyrosine-tripeptide had no effect. Similar results were obtained for an isoleucine-auxotrophic *Streptococcus agalactiae* strain, i.e. the addition of isoleucine-containing dipeptides to CDM depleted of this amino acid was able to restore the growth defect of the bacteria^58^. In summary, these results indicate that tryptophan is the ligand of TrpX.

Besides, low affinity-binding of tryptophan is sufficient in nutrient-rich medium, but not under nutrient-limited conditions. Therefore, *trpX* is essential for survival of *S. suis* in different host compartments such as the brain or the blood^24^. In general, blood is a rich source of glucose and free amino acids. Porcine serum contains approximately 12.5 mg/L tryptophan^59^. Hence, the auxotrophy of *S. suis* might be an evolutionary adaptation to the nutrient supply in the host^60^. However, without TrpX, *S. suis* is not able to import enough tryptophan to survive under these conditions. In accordance with the study on *S. pyogenes*^54^, these results highlight the importance of high-affinity binding transporters during in vivo infections.

RT-PCR and 5′ RACE revealed that the genes encoding *trpXYZ* are co-transcribed with a single promoter upstream of *trpX*. Thus, TrpXYZ is organized as an operon, which is a typical feature of ABC transporters^47^. As ABC transporters are important fitness-associated factors and, as such, may contribute to virulence, their expression is strictly regulated^11^. Many of these transporters are not constitutively expressed but induced under nutrient-limited conditions^10^, which can be found in the host. While they are often regulated at the transcriptional level of gene expression, the activity of the transporters can be controlled as well^13^. There are many different mechanisms of how this regulation is achieved. In *E. coli* and *Salmonella* Typhimurium the maltose/maltodextrin transporter directly regulates transcription^13^. However, data on other bacterial species indicate that also osmoregulation^61^, phosphorylation^62^ or T-box riboswitches^16^ play a role. Bioinformatics analysis suggested transcription regulation of *trpXYZ* via a T-box riboswitch. The T-box riboswitch monitors the aminoacylation status of the tRNA to induce expression of regulated downstream genes^18^. Binding of the uncharged cognate tRNA stabilizes the antiterminator conformation and leads to the transcription of the downstream gene, whereas binding of charged tRNA results in the formation of the terminator structure and subsequent transcription termination^17^. In bacteria, intrinsic terminators are usually formed by a G/C-rich hairpin structure followed by a thymine stretch of approximately seven to nine thymines (T) in the DNA or Us in the transcribed RNA^48,49^. In the *trpX* T-box the terminator loop is followed by a stretch of only four U residues. Although most of the terminators include six or more U residues, this is not the only characteristic for a successful termination. D'Aubenton Carafa et al*.* showed that a small loop as well as a high GC content of the hairpin can compensate for a shorter U-stretch and vice versa^49^. Furthermore, especially the proximal U residues are important for the formation of the hairpin structure whereas the distal ones are required for the pausing of the elongation complex^48^. To conclude, the four U-stretch of the *trpX* terminator is sufficient for terminating the transcription. The *trpX* T-box comprises 461 nt and is relatively large in size compared to other T-boxes^21^. However, a T-box with a similar size (440 nt) which is involved in methionine biosynthesis has been found in *Staphylococcus aureus*^63^.

Growth in CDM with low tryptophan concentrations resulted in mRNA induction of *trpX* as well as an increased activity of a *gfp*-reporter construct. In contrast, there was no mRNA induction or reporter activation during normal growth conditions. Consequently, *trpXYZ* is not constitutively expressed, but upregulated under tryptophan-limited conditions. This effect is not dose-dependent but seems to be switched ON/OFF when tryptophan levels drop below a certain threshold. Accordingly, T-box regulators have been described as ON switches activating gene expression under nutrient-limited conditions^64^. In our experiments, the lower reporter activation at 0.25 mg/L tryptophan might be due to limitations in protein biosynthesis due to a lack of tryptophan.

Similar results were described for a methionine auxotrophic *Lactobacillus lactis* strain. Hernandez-Valdes et al. reported that in bacteria encountering methionine starvation the T-box riboswitch is induced which leads to an increased expression of a high-affinity methionine transporter^65^. Interaction of stem I with the tRNA plays an important role in binding and ligand recognition^50^. Therefore, we deleted the entire stem I region to investigate its function in ligand binding. In the reporter assay the strain lacking the stem I domain showed no transcript activation at low tryptophan concentrations. Moreover, Hernandez-Valdes et al. investigated several mutations of highly conserved T-box domains and showed that mutagenesis of the key residues of the T-box led to its malfunction since uncharged tRNA was not able to bind anymore^65^. In our study we also mutated the specifier sequence of the *trpX* T-box located in stem I to inhibit the base-pairing with the cognate tRNA. This mutation is of special interest^66^, as this sequence is the primary ligand-specificity determinant^17^ and specific to one amino acid only^18^. Mutations of the specifier sequence were shown to be sufficient to switch the ligand specificity of the T-box^18^. Accordingly, we observed that the T-box with the mutated specifier sequence did not respond to changes in tryptophan concentration anymore. Besides, we mutated the T-box sequence in the antiterminator bulge that base pairs with the acceptor arm of the uncharged tRNA^17,23^. A single mutation of this conserved loop region resulted in a non-inducible *trpX* T-box riboswitch at low tryptophan concentrations which has also been observed for an analogous mutant in the *tyrS* T-box of *Bacillus subtilis*^23^.

The T-box riboswitch analyzed in our study induced expression of *trpX* under tryptophan-limited conditions. A recent study by Zhang et al*.* revealed that a complete deletion of the T-box riboswitch sequence in a serine riboswitch resulted in high transcription of the regulated gene regardless of the serine concentration^67^. Accordingly, the complete deletion of the *trpX* T-box riboswitch sequence also resulted in a constitutively expressed reporter with reduced GFP amounts at the lower concentration possibly due to limited tryptophan availability. Similar results were found in *S. aureus* where the deletion of the antiterminator and terminator stretch led to an activated transcription independent of methionine concentration. Therefore, the T-box riboswitch mechanism depends on an effective termination site^63^.

Interestingly, we identified a T-box riboswitch in the homologous protein of *S. pneumoniae* but not in *S. pyogenes*. The absence of a T-box regulatory element in *S. pyogenes* has already been described in an earlier study^54^. Nevertheless, T-box riboswitches are widespread in Gram-positive bacteria and often participate in the regulation of operons linked to amino acid synthesis or transport, especially in *Firmicutes*^17,68^. However, there are also other mechanisms that have been described for sensing changing levels of uncharged tRNA^69^. For tryptophan operons this comprises a tryptophan translating ribosome in *E. coli*^70^ as well as a tryptophan-activated RNA-binding protein in *B. subtilis*^71^. Further studies are required to clarify whether the T-box riboswitch is specific for *S. suis* and *S. pneumoniae* or whether it is a common scheme in orthologues.

## Conclusions

In summary, our study showed that TrpX is part of an ABC transporter involved in tryptophan uptake where TrpX predictably has a role in substrate binding. On the chromosome, the *trpX* gene is organized as an operon together with two genes coding for a transposase (TrpY) and an ATPase (TrpZ).The operon is transcriptionally regulated by a tryptophan T-box riboswitch. Since *S. suis* strain 10 is auxotrophic for tryptophan, TrpX is crucial for metabolic adaptation and growth of the pathogen in environments with tryptophan limitations as those encountered during infection. As TrpX is predicted to be the substrate-binding protein, future studies are required to elucidate the role of amino acids, which were predicted to be important in substrate binding and specificity during bacterial growth. In addition, the other components of this transporter, TrpY and TrpZ, need to be characterized in more detail.

Due to the relevance of TrpX during infection and for the survival of *S. suis* in porcine blood^24^, it might be an interesting target for antimicrobial drugs or vaccines. In previous studies not only ABC transporters^9^, but also T-box riboswitches have been proposed as interesting targets for the development of new therapeutics^72,73^. Finally, due to its tryptophan auxotrophy *S. suis* as well as the plasmids and mutants generated in this study represent an excellent model to study regulation and biological function of these transporters in detail to better understand metabolic adaptation of bacterial pathogens in different host environments.

## Supplementary Information

Supplementary Information.

## Data Availability

All data are contained within the manuscript except for the modified T-box scan code which is available in the GitHub repository at https://github.com/AndreasNerlich/Streptococcus_suis_TrpX.
