# Supplementary material for: Streptococcus suis TrpX is part of a tryptophan uptake system, and its expression is regulated by a T-box regulatory element
Source: Sci Rep. 2022 Aug 17;12:13920. doi: 10.1038/s41598-022-18227-3 (PMC9382623; doi:10.1038/s41598-022-18227-3)
Supplement: Supplementary file 1 — Supplementary Information. [file 41598_2022_18227_MOESM1_ESM.pdf]

***Streptococcus suis* TrpX is part of a tryptophan uptake system and its expression is regulated by a T-box regulatory element**

Muriel Dresen[1]*, Desirée Schaaf[1], Jesús Arenas[2], Astrid de Greeff[3], Peter Valentin-Weigand[1#], Andreas Nerlich[1,4#]

[1] Institute for Microbiology, University of Veterinary Medicine Hannover, Germany

[2] Unit of Microbiology and Immunology, Faculty of Veterinary, University of Zaragoza, Zaragoza, Spain

[3] Wageningen Bioveterinary Research, part of Wageningen University and Research, Lelystad, The Netherlands

[4] Current address: Veterinary Centre for Resistance Research (TZR), Freie Universität Berlin, Berlin, Germany

*Correspondence: muriel.dresen@tiho-hannover.de; Tel.: +49-511-856-7776

[#] Shared last authors.

Supplementary Information

	Purpose	Primer	Sequence (5`- 3`)
**10Δ*trpX* mutant**	First PCR	Fssu1307-L	GACTGATGGATACCGTATAATC
		Rssu1307-L	ACGAACGAAAATCGACCTGCATTCATTCTCCTCGTAAAATAC
	Second PCR	Fssu1307-R	TTAGAAAACAATAAACCCTTGCATGAGATAAGAGAAAAGGAA
		Rssu1307-R	TAGAAAATCGCTCCGACAAC
	Amplification of Spec cassette	Fspec	GCAGGTCGATTTTCGTTCGT
		Rspec	ATGCAAGGGTTTATTGTTTTCTAA
**10 cTrpX mutant**	Amplification of *SSU1307*	pGA14_1307_IVA_for	CCAAGGCCAGTGTGAATTCGGCAGTCTAACTAGATATAG
		pGA14_1307_IVA_rev	CTTGGCAGGTACCTTATTTGTATAGTCTCTTATTCGATAACCTTGC
	Plasmid PCR	pGA14_CM_IVA_rev	GAATTCACACTGGCCTTGGTTAAG
		pGA14_CM_IVA_for	ACTATACAAATAAGGTACCTGCCAAG
**Colony PCR**	Amplification of *SSU1307*	SSU1307_for	TGTTATGGTAGTTGCTGCTTTG
		SSU1307_IVA_rev	TGATATGCCTCCTAAATTCTTATTCGATAACCTTGCTTGC
**qRT-PCR**	Amplification of *SSU1307*	SSU1307_for_qRT	TGCACAAGGTCTTGCCAATG
		SSU1307_rev_qRT	AGAGAAACTGCGTCTGCTAC
	Amplification of *dnaH*	dnaH_for	GTCGCAAAACGGCTCATACT
		dnaH_rev	AGCCTTAGTCAGGACCAGCA
**Operon Analysis**	5'RACE	Template Switching Oligo (TSO)	GCTAATCATTGCAAGCAGTGGTATCAACGCAGAGTACATrGrGrG
		TSO_spec_PCR	CATTGCAAGCAGTGGTATCAAC
		SSU1307_GSP_5'RACE_I	ACTTGGGATTGATCACCCTCAGCATTC
		SSU1307_GSP_5'RACE_II	CCGTTGTGGTATAACCACCTTCTTCAAGTC
	Co-transcription PCR	TrpX_for	CCGCACACCTTACAGTGCAAC
		TrpX_1_for	GAGCCTGTGGTGCTGAGAAC
		TrpY_rev	TTCTTCAGCCGTTTGGTC
		SSU1307_GSP_5'RACE_II	CCGTTGTGGTATAACCACCTTCTTCAAGTC
**cRACE**	cDNA synthesis	cRACE_GSP_1_rev	GCTATACCGCCAGTATAAGC
	1st PCR	cRACE_P1_for	GTTCGGTCCGCACACCTTAC
		cRACE_P2_rev	ACTAGGCAGTCGGGTGAAGG
	2nd PCR	cRACE_N1_for	AAGTGAGGTGGCACCGTGTC
		cRACE_N2_rev	CGGCAAGCACGACTAACGAC
**Mut_ Specifier Sequence**	T-box Mutagenesis PCR	Specifier_Seq_Mut_for	CGGAGTTTTATGAAATACGCTGATGTGCATTTATGTC
		Specifier_Seq_Mut_rev	ATTTCATAAAACTCCGCCACCCGTTCTCAGC
**Mut_ T-box sequence**		T-box_sequence_Mut_for	CATAAGTGAGGTGACACCGTGTCATTGACGC
		T-box_sequence_Mut_rev	CACCTCACTTATGGGAATTGTCTAGAATCCTC
**Stem I Deletion**		Stem_I_Del_for	CCGTATAATCATCTGTAATCGTGCTTGCCGTACC
		T-box_deletion_rev	TTACAGATGATTATACGGTATCCATCAG

**T-box Deletion**		T-box_deletion_for	CCGTATAATCATCTGTAATTTTCCATTGTCTTC
		T-box_deletion_rev	TTACAGATGATTATACGGTATCCATCAG

Suppl. Table S1. Primers used in the present study.

Compound	Concentration (mg/L)
L-Alanine	100
L-Arginine	100
L-Aspartic acid	100
L-Asparagine	100
L-Cysteine	100
L-Cystine	100
L-Glutamic acid	100
L-Glutamine	200
Glycine	100
L-Histidine	100
L-Isoleucine	100
L-Leucine	100
L-Lysine	100
L-Methionine	100
L-Phenyalanine	100
L-Proline	100
Hydroxy-L-Proline	100
L-Serine	100
L-Threonine	200
L-Tryptophan	varied
L-Tyrosine	100
L-Valine	100
p-Aminobenzoic acid (vitamin B10)	0.4
Biotin (vitamin B7)	0.4
Folic acid (vitamin B9)	1.6
Niacinamide (vitamin B3)	2
β-Nicotinamide adenindinucleotide (NAD)	5
Pantothenate calcium salt (vitamin B5)	4
Pyridoxal (vitamin B6)	2
Pyridoxamine dihydrochloride	2
Riboflavin (vitamin B2)	4
Thiamine hydrochloride (vitamin B1)	2
Vitamin B12	0.2
Adenine	40
Guanine	40
Uracil	40
K_2HPO_4	400
KH_2PO_4	2,000
NaH_2PO_4 x H_2O	6,400
Na_2HPO_4 x $2{\cdot}H_2O$	14,700
$NaC_2H_3O_2$ x $3{\cdot}H_2O$	5,400
$NaHCO_3$	5,000
$FeSO_4$ x $7{\cdot}H_2O$	10
$Fe(NO_3)_2$ x $9{\cdot}H_2O$	2
$MgSO_4$ x $7{\cdot}H_2O$	682
$MnSO_4$	10
$CaCl_2$ x $2{\cdot}H_2O$	10
Glucose	50 mM

Suppl. Table S2. Composition of chemically defined medium (CDM). CDM used for growth experiments in this study according to Willenborg *et al.* [1].

Promotor	Promotor position	LDF	-10 box	-10 box position	-10 box score	-35 box	-35 box position	-35 box score
1	-676	8,17	CCGTATAAT	-691	76	TTGACA	-711	66
2	-45	4,38	TGGGATAAT	-60	58	ATGAAA	-78	30
3	-346	1,07	ACTTAAAAC	-361	29	TAGCCA	-385	24

Suppl. Table S3. Results promoter prediction. The promotor of *S. suis trpX* was predicted with Softberry BPROM promotor prediction [2]. The program found three different promoters whereof the one with the highest score is located -676 bp upstream of the *trpX* ATG codon. Position of the promoter, the -10 box and the -35 box with corresponding scores are shown. The LDF value represents the threshold.

	S. suis	***S. pneumoniae***	***S. pyogenes***
**Locus**	SSU1307	SP1069	SPy1016
**T-box detected**	Yes	Yes	No
**E-value**	1.4e-12	8.4e-28	/
**Score**	45.9	107.3	/
**CM_Accuracy**	0.87	0.92	/
**AA Family**	T-box Trp	T-box Trp	/
**Specifier region**	AUGGG	UUGGG	/
**Specifier**	UGG	UGG	/
**T-box sequence**	UGGC	UGGU	/

Suppl. Table S4. Results T-box scan. *S. suis trpX* and the homologous genes of *S. pneumoniae* and *S. pyogenes* were analyzed with T-box scan [3]. The E-value (expectation value) represents the statistical significance of the hit. The score is the log-odds score for the hit. The CM_Accuracy value represents the average posterior probability of the aligned target sequence residues. For more detailed information see the INFERNAL user guide (http://eddylab.org/infernal/Userguide.pdf). The software predicted a tryptophan (trp) T-box for *S. suis* and *S. pneumoniae*. Nucleotide sequences of the specifier region, the specifier sequence and the T-box sequence are shown.

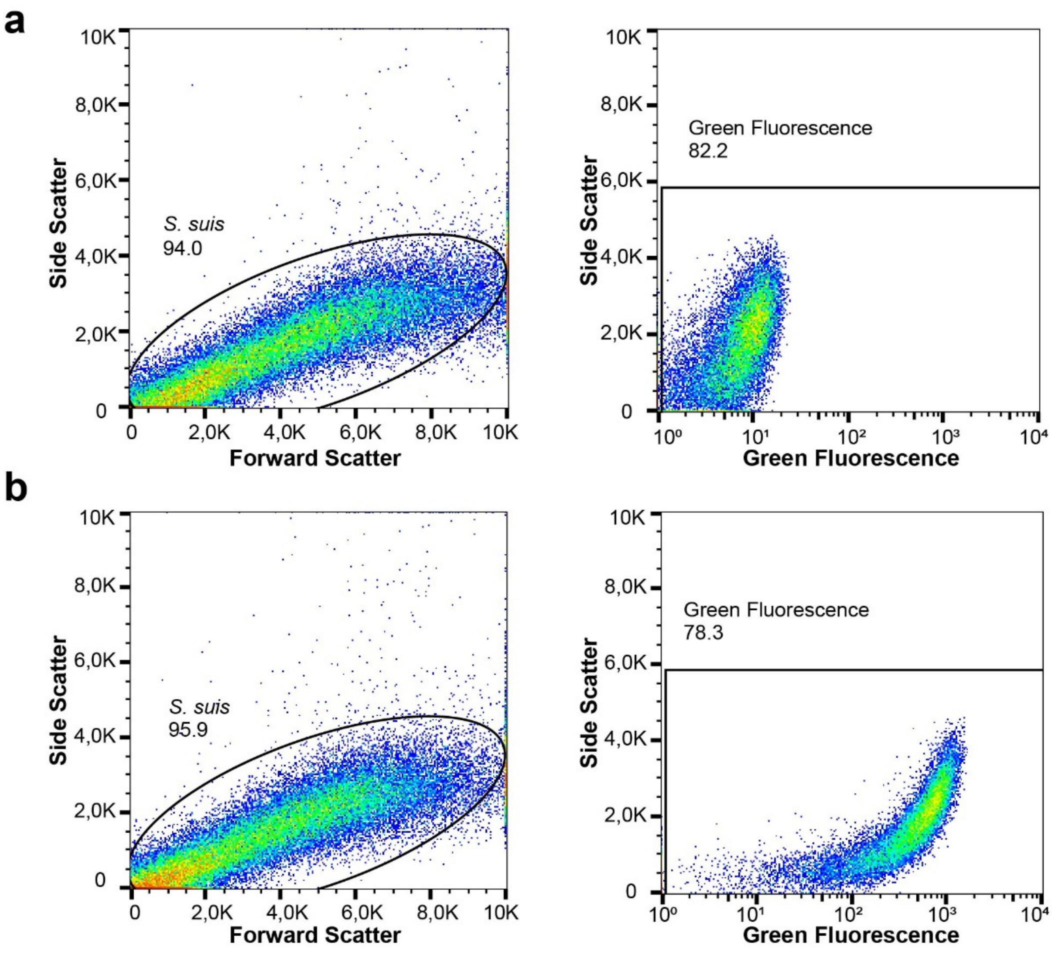

Suppl. Figure S1. Gating strategy for flow cytometry analysis with FlowJo. Events were gated for *S. suis* using forward and side scatter. The resulting population was gated for green fluorescence. In (**a**) the gating of *S. suis* strain 10 in CDM with 5.0 mg/L and in (**b**) the gating of 10::*trpXYZ*-prom-*gfp* in CDM with 0.5 mg/L tryptophan is shown.

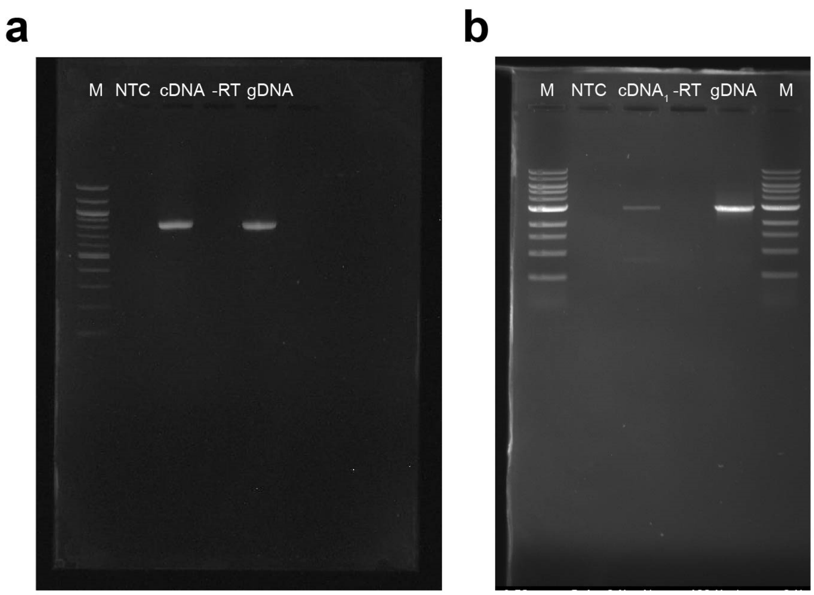

Suppl. Figure S2. Original gels from Fig. 3c. Gel number 1 is shown in (**a**) and gel number 2 in (**b**). NTC, non-template control; cDNA, standard RT-PCR reaction using reverse transcribed RNA; -RT, negative control in which no reverse transcriptase was added to the RT reaction; gDNA, positive control in which genomic DNA was used as a template in the RT-PCR.

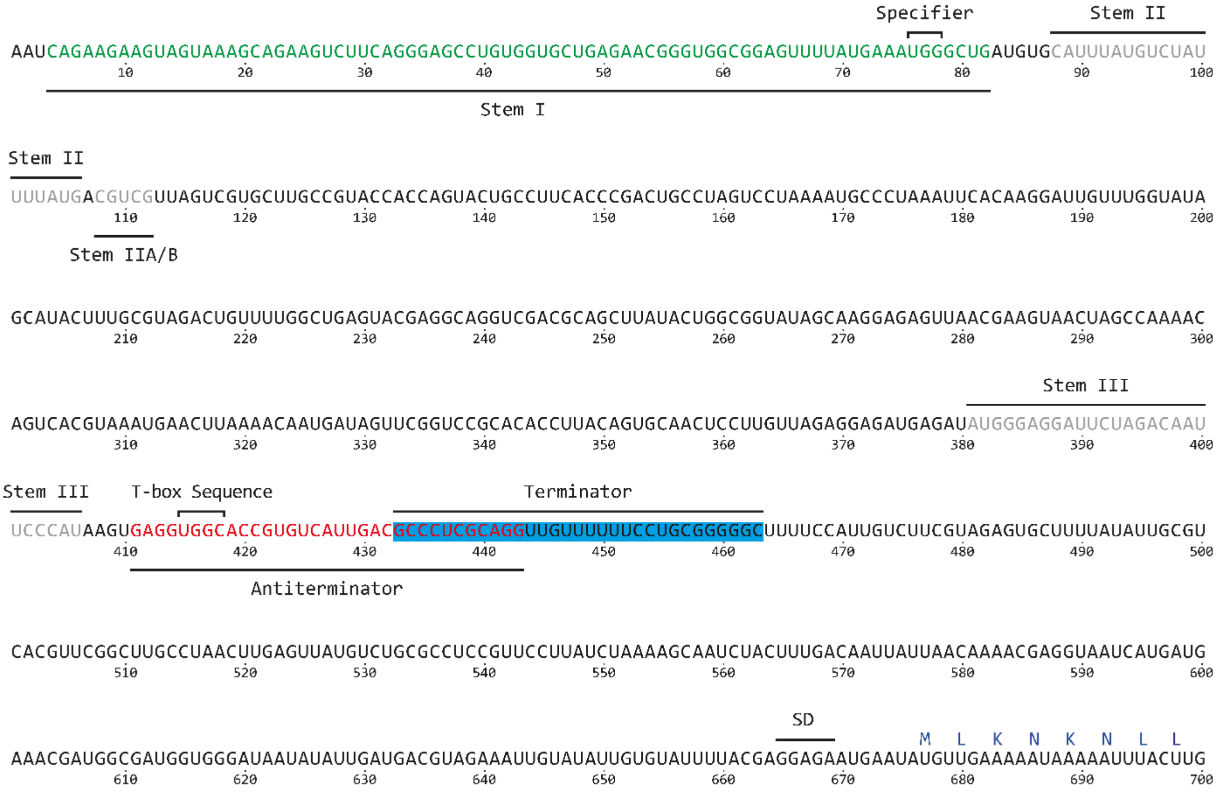

Suppl. Figure S3. Nucleotide sequence of the 5´-flanking region and beginning of the coding region of the *trpX* mRNA according to the P1/7 genome. Stem I with specifier sequence (green), stem II, IIA/B and III (grey), partially overlapping antiterminator with T-box sequence (red) and terminator sequence (blue colored background) as well as Shine-Dalgarno (SD) sequence are indicated. Start of the coding region is indicated by amino acids (blue, single letter code).

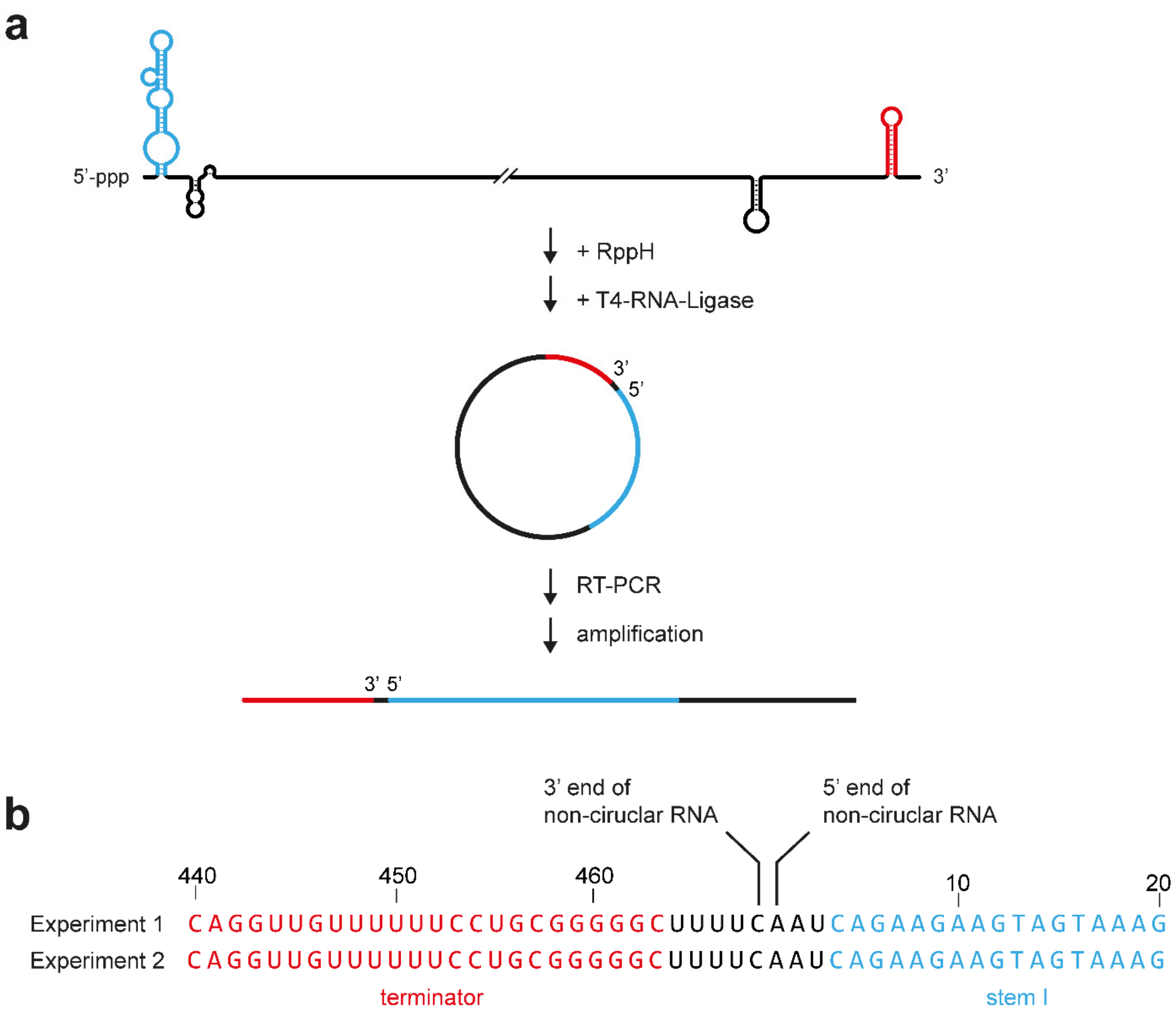

Suppl. Figure S4. cRACE. (**a**) *S. suis* RNA isolated at 5 mg/L Trp was treated with RppH to remove pyrophosphate and ligated with T4-Ligase. The circularized RNA was used for cDNA synthesis and subsequent PCR amplification. The amplicon of the nested PCR was analyzed by sequencing. Sequencing results are shown in (**b**). The end of the sequence of the terminator is shown in red and stem I is shown in blue. 3' and 5' end are indicated.

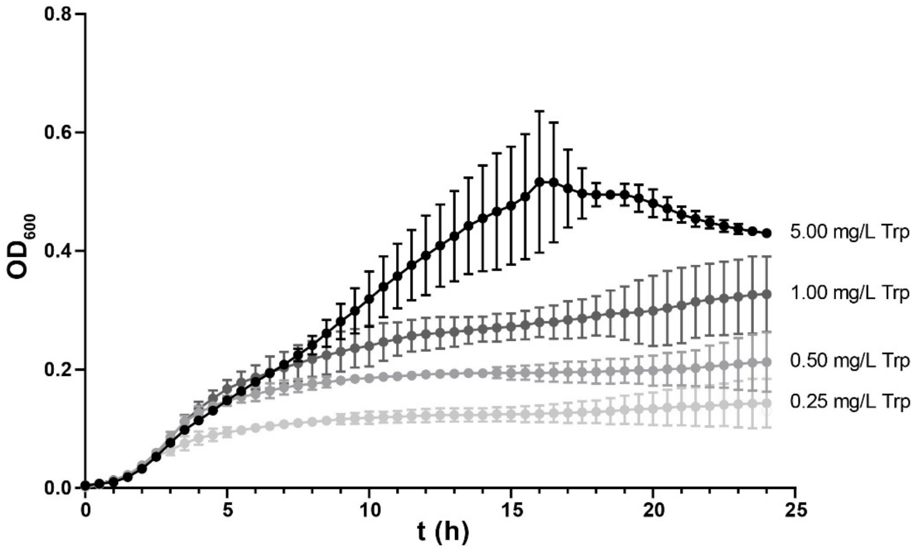

Suppl. Figure 5. Growth of *S. suis* strain 10 in CDM with reduced tryptophan concentrations. *S. suis* strain 10 (grey) was grown in CDM supplemented with different tryptophan concentrations for 24 h. Optical density at 600 nm (OD600) was determined every 30 minutes for 24 h. Data are presented as mean ± SD of two independent experiments.

References

1 Willenborg, J. *et al.* Characterization of the pivotal carbon metabolism of Streptococcus suis serotype 2 under ex vivo and chemically defined in vitro conditions by isotopologue profiling. *J Biol Chem* **290**, 5840-5854, doi:10.1074/jbc.M114.619163 (2015).

2 Solovyev V, S. A. in *Metagenomics and its Applications in Agriculture* Ch. Biomedicine and Environmental Studies 61-78 (Nova Science Publishers, 2011).

3 Marchand, J. A., Pierson Smela, M. D., Jordan, T. H. H., Narasimhan, K. & Church, G. M. TBDB: a database of structurally annotated T-box riboswitch:tRNA pairs. *Nucleic Acids Res* **49**, D229-d235, doi:10.1093/nar/gkaa721 (2021).
